# A Broad‐High Temperature Ceramic Capacitor with Local Polymorphic Heterogeneous Structures

**DOI:** 10.1002/advs.202409814

**Published:** 2024-10-30

**Authors:** Binglong Zheng, Ying Lin, Haibo Yang, Hongmei Jing, Hu Nan, Yifei Wang, Fang‐Zhou Yao, Minquan Wang, Qibin Yuan

**Affiliations:** ^1^ Shaanxi Key Laboratory of Green Preparation and Functionalization for Inorganic Materials School of Materials Science and Engineering Shaanxi University of Science and Technology Xi'an 710021 China; ^2^ School of Physics and Information Technology Shaanxi Normal University Xi'an 710119 China; ^3^ School of Microelectronics Faculty of Electronic and Information Engineering Xi'an Jiaotong University Xi'an 710049 China; ^4^ State Key Laboratory for Mechanical Behavior of Material School of Materials Science and Engineering Xi'an Jiaotong University Xi'an 710049 China; ^5^ Research Center for Advanced Functional Ceramics Wuzhen Laboratory Jiaxing 314500 China; ^6^ School of Electronic Information & Artificial Intelligence Shaanxi University of Science and Technology Xi'an 710021 China

**Keywords:** ceramic capacitors, high‐temperature energy storage, lead‐free, polymorphic heterogeneous structure

## Abstract

Crafting high‐performance dielectrics tailored for pulsed power capacitors, in response to the escalating demands of practical applications, presents a formidable challenge. Herein, this work introduces a novel lineup of lead‐free ceramics with local polymorphic heterogeneous structures, defined by the formula (1‐*x*)[0.92BaTiO_3_‐0.08Sr(Mg_1/2_Ti_3/4_)O_3_]‐*x*(Na_0.5_Bi_0.5_)TiO_3_ (BT‐SMT‐*x*NBT). This innovative multi‐scale synergistic strategy, spanning from the atomic to grain scale, yields materials with a giant recoverable energy density (*W*_rec_) of 10.1 J·cm^−3^ and an impressive energy efficiency (*η*) of 95.0%. The integration of linear end elements SMT can significantly mitigate the polarization hysteresis while concurrently boosting the breakdown strength, thus enhancing overall energy efficiency. Furthermore, the inclusion of NBT with high polarization serves to amplify domain size, thereby reinforcing the electric field‐induced polarization. This addition also stimulates the creation of polymorphic heterostructures, where tetragonal and rhombohedral nanodomains coexist, as validated by aberration‐corrected transmission electron microscopy. Notably, the BT‐SMT‐0.2NBT ceramics have demonstrated outstanding high‐temperature energy storage capabilities, with a *W*_rec_ of 7.2 J·cm^−3^ and an *η* of 92.2% at 150 °C, along with remarkable broad‐temperature stability (Δ*W*_rec_, Δ*η* ≤ 4.0%, ≈20–150 °C). These achievements in this work propel the field toward more practical and durable solutions of energy storage dielectrics.

## Graphical abstract

Developing high performance dielectrics for pulsed power capacitors poses significant challenges, especially considering the rising energy demands and increasing environmental concerns. Enhancing BaTiO_3_‐based lead‐free energy storage ceramic for miniaturization and high‐temperature applications is essential. This study introduces multi‐scale optimization strategies to improve their design and performance for efficient energy management.

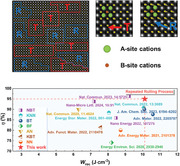

## 1 Introduction

Dielectric capacitors have risen to prominence in the realm of pulsed power devices, attributable to their exceptional power density and ultrafast charging and discharging capabilities. However, the limited energy density of dielectric capacitors hampers their widespread applicability.^[^ ^1^, ^2^ ^]^ To this end, a surge of research endeavors has been channeled into the development of dielectric materials that are not only reliable and efficient but also boast augmented energy densities. In the current landscape, the cadre of dielectric materials harnessed for energy storage encompasses a spectrum of options, including polymer‐based thick films, thin films, and ceramics.^[^ ^3^, ^4^, ^5^ ^]^ Among them, ceramic‐based materials have garnered considerable attention, largely due to their commendable high‐temperature stability and diminished dielectric loss.^[^ ^6^, ^7^, ^8^ ^]^ The quest for environmentally benign alternatives have steered recent research efforts toward lead‐free ceramics, with notable examples such as BaTiO_3_ (BT),^[^ ^9^, ^10^ ^]^ (Na_0.5_Bi_0.5_)TiO_3_ (NBT),^[^ ^11^, ^12^, ^13^ ^]^ (K_0.5_Na_0.5_)NbO_3_ (KNN),^[^ ^14^, ^15^ ^]^ and NaNbO_3_ (NN)^[^ ^16^, ^17^ ^]^‐based ceramics. Despite the advancements, it is acknowledged that lead‐free ceramics must still surpass traditional lead‐based ceramics in terms of recoverable energy density (*W*_rec_) and energy efficiency (*η*) to meet the stringent criteria for integration and miniaturization in modern applications. This necessitates ongoing optimization and innovation in material design to unlock their full potential and establish them as viable contenders in the domain of dielectric capacitors.

The quest for superior dielectric capacitors is underpinned by the well‐established understanding that a robust maximum polarization (*P*_max_), minimal remnant polarization (*P*_r_), and elevated breakdown strength (*BDS*) are pivotal in the pursuit of exceptional *W*_rec_ and *η*.^[^ ^18^, ^19^, ^20^ ^]^ In the relentless drive to enhance the energy storage performance of lead‐free ceramics, a multitude of strategies have been explored, reflecting a concerted effort to fine‐tune these critical parameters. From the intricate nanoscale to the broader macroscale, the reported strategies are aimed at curtailing polarization hysteresis, alleviating polarization saturation and bolstering the breakdown strength.^[^ ^9^, ^15^, ^21^, ^22^, ^23^ ^]^ Notably, advancements have been marked by the achievement of *W*_rec_ surpassing 10.0 J·cm^−3^ in BT‐,^[^ ^9^, ^24^ ^]^ KNN‐,^[^ ^14^ ^]^ and NBT‐based^[^ ^12^, ^25^, ^26^ ^]^ lead‐free ceramics, leveraging mechanisms such as superparaelectric state, high‐entropy design. However, it is essential to recognize that the energy loss during capacitor operation predominantly dissipates as heat, which can inevitably escalate the ambient temperature, thereby escalating the dielectric loss and heightening the risk of thermal breakdown, resulting in a precipitous drop in energy storage performance.^[^ ^14^, ^15^, ^26^ ^]^ Consequently, there is an urgent need to innovate lead‐free ceramic capacitors that can deliver ultra‐high energy density and maintain high efficiency over a broad operating temperature range. The establishment of polymorphic polar nano‐microstructures within ceramics is conducive to bestowing structural stability at high temperatures. This stability is attributed to the pronounced local heterogeneity in the ordered‐disordered system, which exerts a “pinning” effect and thereby hindering local structural changes induced by thermal vibrations of the atoms, and thus ensuring the stability of the energy storage across a diverse temperature spectrum.^[^ ^11^, ^27^, ^28^ ^]^ BT, with an uncomplicated crystal structure and room temperature T‐phase configuration, has emerged as a key player in the commercial technology sector, particularly in its application as a high permittivity dielectric in multilayer ceramic capacitors.^[^ ^18^, ^29^ ^]^ However, pristine BT ceramics are still confronted with tremendous challenges, as they display significant polarization hysteresis and dielectric constant instability in a broad temperature range, impeding their suitability for the evolving demands of power electronic devices.^[^ ^30^, ^31^ ^]^ In light of these considerations, the future trajectory of BT‐based ceramic capacitors is directed toward achieving high *η* and maintaining excellent energy storage performance over a broad and high temperature range, positioning these materials as key contenders in the vanguard of dielectric technology.

Here, we rationally designed a series of lead‐free ceramics, denoted as (1‐*x*)[0.92BaTiO_3_‐0.08Sr(Mg_1/2_Ti_3/4_)O_3_]‐*x*(Na_0.5_Bi_0.5_)TiO_3_ (BT‐SMT‐*x*NBT), featuring local polymorphic heterogeneous structures through a multi‐scale synergistic strategy. At the atomic scale, the BT‐SMT‐*x*NBT lead‐free ceramics demonstrate remarkably low energy loss and an enhanced breakdown strength by introducing linear end elements SMT.^[^ ^32^, ^33^ ^]^ Despite this, the introduction of the linear end‐element poses a trade‐off, potentially leading to a reduction in spontaneous polarization for the system. To counteract this, we have strategically integrated NBT, known for its substantial R‐phase ferroelectric domains (large saturation polarization). This addition serves to augment the electric field‐induced polarization behavior and fosters the emergence of the polymorphic heterostructure, where rhombohedral and tetragonal polar nano regions coexist.^[^ ^34^, ^35^ ^]^ Previous studies have demonstrated that the energy barriers between polarized states with varying orientations are considerably reduced in polymorphic nanodomain structures compared to those of single‐phase nanodomains, leading to high polarization and diminished hysteresis.^[^ ^36^, ^37^ ^]^ Furthermore, at the grain scale, the repeated rolling processing (RRP) method is employed to craft ceramic samples with reduced grain sizes, thereby bolstering the breakdown strength of the ceramics.^[^ ^38^, ^39^ ^]^ The integration of polymorphic heterostructure design with process optimization within our synergistic strategy holds tremendous potential for achieving exceptional broad‐high temperature energy storage performance.

## 2 Results and Discussion

### 2.1 Energy Storage Performance of BT‐SMT‐NBT Ceramics

**Figure 1:**
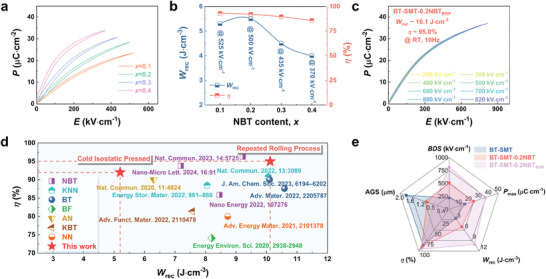
Energy storage performance of BT‐SMT‐*x*NBT ceramics. a) *P*‐*E* loops and b) the calculated *W*_rec_ and *η* at *E*_b_ for different compositions. c) *P*‐*E* loops of the BT‐SMT‐0.2NBT_RRP_ composition under different electric fields. d) Comparisons of *W*_rec_ and *η* (≈RT) between our work with some recently reported lead‐free bulk ceramics. e) Comparison of *BDS*, *P*_max_, *W*_rec_, *η* and AGS values among BT‐SMT, BT‐SMT‐0.2NBT and BT‐SMT‐0.2NBT_RRP_ ceramics.

The energy storage capacity of a material is readily discernible through an examination of its polarization behavior when subjected to the same electric field. At an electric field of 200 kV·cm^−1^ (Figure S1, Supporting Information), the BT‐SMT exhibits commendably low loss, but its *P*_max_ peaks at a modest 8 µC·cm^−2^, hindering the achievement of superior *W*_rec_. As the incorporation of NBT incrementally increased, there is a marked improvement in *P*_max_, a result of the additional orbital hybridization between Bi 6*s* and O 2*p*, albeit with a modest rise in polarization hysteresis. The *W*_rec_ and *η* (**Figure** **1**b) of the BT‐SMT‐*x*NBT compounds are derived from their unipolar *P*‐*E* loops (Figure 1a). While the BT‐SMT‐0.1NBT ceramic achieves a respectable efficiency of 93.6%, the *W*_rec_ is mere 4.1 J·cm^−3^ due to the unsatisfying polarization saturation. Optima results are obtained by introducing 0.2 mol of NBT, yielding *W*_rec_ of 5.2 J·cm^−3^ and *η* of 92.5%, respectively, at an electric field of 500 kV·cm^−1^. It is noteworthy, however, that when *x* exceeds 0.2, the domain size inflates excessively, resulting in a decline in efficiencies below the 90.0% threshold. In the pursuit of augmenting the *W*_rec_ of BT‐SMT‐0.2NBT ceramics, the thickness and grain size of the ceramics were refined through RRP, thereby escalating the breakdown strength. The room temperature unipolar *P*‐*E* loops of the BT‐SMT‐0.2NBT_RRP_ ceramic, measured from 200 kV·cm^−1^ to the critical electric field of 820 kV·cm^−1^ at 10 Hz (Figure 1c), reveal an ultra‐low *P*_r_ of 0.97 µC·cm^−2^. This finding underscores the electric field‐insensitivity of *P*_r_ value and highlights exceptional *η* of 95.0%, along with an impressive *W*_rec_ value of 10.1 J·cm^−3^. The reliability of the *BDS* is validated through Weibull distribution analysis, as shown in Figure S2, Supporting Information. The Weibull modulus (*β*) of the BT‐SMT‐0.2NBT_RRP_ ceramic is 18.2, indicating that the sample exhibits high reliability. The calculated statistical *BDS* is 823.3 kV·cm^−1^, closely corresponding to the *BDS* obtained in the *P*‐*E* loop. A comparative analysis with recently reported energy storage ceramics (Figure 1d and Table S1, Supporting Information) underscores the competitive edge of BT‐SMT‐0.2NBT_RRP_ ceramic in energy storage performance at RT,^[^ ^9^, ^10^, ^11^, ^14^, ^16^, ^23^, ^40^, ^41^, ^42^, ^43^, ^44^ ^]^ attributed to enhanced electric field‐induced polarization and downsized average grain sizes (AGS, from 1.76 to 0.70 µm, see Figure 1e). Compared to the BT‐SMT‐0.2NBT ceramics, the BT‐SMT‐0.2NBT_RRP_ ceramics are thinner, which contributes to a notable reduction in sintering temperature and time, ultimately leading to finer grains and a denser microstructure.^[^ ^38^, ^45^ ^]^

### 2.2 Broad‐High Temperature Stability for Practical Application

**Figure 2:**
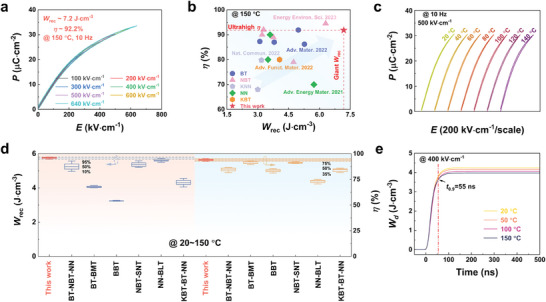
a) Calculated *P*‐*E* loops for the BT‐SMT‐0.2NBT_RRP_ ceramic composition at 150 °C. b) Comparison of *W*_rec_ and *η* of the reported state‐of‐the‐art high‐temperature dielectric materials. c) Temperature‐dependent *P*‐*E* loops at 500 kV·cm^−1^. d) Comparison between the temperature‐variant energy storage performance of the BT‐SMT‐0.2NBT_RRP_ ceramic and other reported ceramics. e) Time dependence of discharge energy density under different temperatures.

Ceramic capacitors are frequently deployed in intricate environments that necessitate both a broad operating temperature range and excellent high‐temperature energy storage performance. Therefore, the *P*‐*E* loops of BT‐SMT‐0.2NBT_RRP_ ceramic were collected at 150 °C in this study (**Figure** **2**a). It is noted that a substantial *W*_rec_ of 7.2 J·cm^−3^ is realized in BT‐SMT‐0.2NBT_RRP_ ceramic, coupled with an excellent *η* of 92.2% under a robust breakdown electric field of 640 kV·cm^−1^. The *W*_rec_ and *η* exhibit a modest decline when the ceramic is exposed to 150 °C in comparison to its performance at RT. This performance decrement could be associated with an upsurge in leakage current at higher temperatures, which in turn leads to diminished breakdown strength and efficiency. Despite that, the BT‐SMT‐0.2NBT_RRP_ ceramic outperforms other state‐of‐the‐art lead‐free ceramics in terms of energy storage performance at high temperatures, as highlighted in Figure 2b.^[^ ^9^, ^10^, ^12^, ^14^, ^15^, ^16^, ^17^, ^39^, ^40^, ^41^, ^46^, ^47^, ^48^, ^49^, ^50^ ^]^ Beyond outstanding performance at high temperatures, temperature stability is also a crucial attribute. The variable‐temperature *P*‐*E* loops of the BT‐SMT‐0.2NBT_RRP_ ceramic were measured at an electric field of 500 kV·cm^−1^ (Figure 2c), and the corresponding *W*_rec_ and *η* were calculated (Figure S3, Supporting Information). As temperature ascends, the *P*‐*E* loops exhibit a progressive reduction in *P*_max_ values. This phenomenon could be attributed to the facilitated domain movement at high temperatures, resulting in stronger local fields. To maintain equivalent polarization levels, a higher electric field is necessary to counteract the local fields.^[^ ^49^ ^]^ Despite this, under a high electric field of 500 kV·cm^−1^, the variation of *W*_rec_ and *η* is kept under 4% over the temperature range from 20 to 150 °C. A thorough comparison of temperature stability among recently reported lead‐free ceramics is summarized in Figure 2d.^[^ ^9^, ^10^, ^16^, ^44^, ^46^, ^51^ ^]^ It is evident that simultaneously achieving large *W*_rec_, high *η*, and excellent stability is a formidable challenge. For instance, 0.85K_0.5_Na_0.5_NbO_3_‐0.15Bi(Zn_2/3_Ta_1/3_)O_3_ ceramics demonstrate exceptional temperature stability within the range of 25 to 125 °C at an electric field strength of 400 kV·cm^−1^. Unfortunately, when the temperature exceeds 125 °C, the energy storage performance deteriorates significantly.^[^ ^15^ ^]^ Additionally, BaTiO_3_‐(Na_0.5_Bi_0.5_)TiO_3_‐NaNbO_3_ ceramics exhibit a high energy storage density within the temperature range of room temperature to 150 °C. However, their energy storage density stability is inadequate, showing a variation rate exceeding 5.0%, which restricts their applicability across a broader temperature range.^[^ ^9^ ^]^ In contrast, the BT‐SMT‐0.2NBT_RRP_ ceramic exhibits a stable *W*_rec_ value ranging from 5.7 to 5.9 J·cm^−3^, with a variation of less than 4.0% across the temperature range of 20 to 150 °C. At the same time, an exceptionally high *η* value of 95.4 ± 4.0%, demonstrating thermal stability that surpasses that of other recently reported lead‐free ceramics. Notably, frequency stability is also essential for the practical application of energy storage ceramics. For the BT‐SMT‐0.2NBT_RRP_ ceramic, as illustrated in Figure S4, Supporting Information, the values of *W*_rec_ and *η* at each frequency remain stable at 500 kV·cm^−1^. This underscores that the BT‐SMT‐0.2NBT_RRP_ ceramic exhibits excellent frequency stability. Throughout the frequency range of 1 to 100 Hz, *W*_rec_ and *η* consistently maintain high values, ranging from 5.8 to 6.0 J·cm^−3^ and 94.3% to 96.0%, respectively. Moreover, the assessment of ceramic capacitors for practical energy storage applications should also consider the charging and discharging performance, another crucial factor. The overdamped discharge characteristics of BT‐SMT‐0.2NBT_RRP_ ceramics at varied temperatures were measured at an electric field of 400 kV·cm^−1^, utilizing a custom‐designed load circuit incorporating a 100 Ω resistor, and the regular overdamped oscillation waveforms reflect steady discharge behavior (Figure S5, Supporting Information). In addition, the discharge density *W*_d_, which can be stabilized at ≈4.1 J·cm^−3^, can be efficiently discharged by 90% (*t*_0.9_) within an ultra‐short time of 55 ns (Figure 2e).

### 2.3 Phase Structure and Dielectric Properties

**Figure 3:**
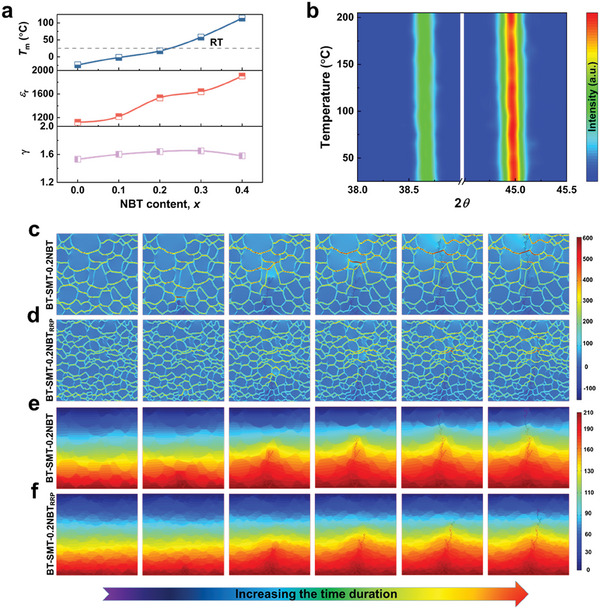
a) *T*_m_, *ε_r_* and *γ* of BT‐SMT‐*x*NBT ceramics at 1 kHz. b) Temperature evolution of XRD patterns for the BT‐SMT‐0.2NBT_RRP_ ceramic from 25 to 205 °C. Electric field distributions and growth paths of the electrical tree for c) BT‐SMT‐0.2NBT and d) BT‐SMT‐0.2NBT_RRP_. Electric potential distributions and growth paths of the electrical tree for e) BT‐SMT‐0.2NBT and f) BT‐SMT‐0.2NBT_RRP_.

Variations in the local phase structure are intimately related to the energy storage performance of ceramic materials. The XRD patterns of BT‐SMT‐*x*NBT ceramics are characterized by high‐intensity, sharp diffraction peaks, indicative of a high degree of crystallinity (Figure S6a, Supporting Information). All the components display a typical perovskite structure without any second phase, suggesting the successful formation of a comprehensive solid solution integrating NBT and BT‐SMT. Raman spectra reveal four distinct vibration modes in the ≈100–1000 cm^−1^ range, and the analysis of these spectral variations can elucidate the evolution of local structures (Figure S6b, Supporting Information). In the low wavenumber region <200 cm^−1^, the observed modes are associated with the vibrations of Ba^2+^, Sr^2+^, Bi^3+^, and Na^+^ cations residing at the A‐site of the lattice. The other three prominent regions in the spectra correspond to the B‐O bond stretching (≈200–400 cm^−1^), the BO_6_ octahedra bond (≈400–700 cm^−1^), and the *A*_1_+*E* overlapping bands (>700 cm^−1^).^[^ ^52^, ^53^ ^]^ It is noteworthy that the broadening of all Raman peaks and increased scattering tendency with the introduction of NBT indicate an increased tilt angle of the BO_6_ octahedron. This phenomenon arises from local disorder induced by multiple elements occupying equivalent lattice sites, which disrupts translational symmetry.^[^ ^54^, ^55^ ^]^ The increased tilt angle of the BO_6_ octahedron might enhance the resistance to torsion under the temperature field. This characteristic improves the temperature stability of polarization in ceramic capacitors, significantly enhancing their high‐temperature energy storage capacity.^[^ ^37^, ^56^ ^]^ To comprehensively assess the dielectric properties across a broad temperature spectrum, the dielectric constant (*ε*_r_) and dielectric loss (tan*δ*) of BT‐SMT‐*x*NBT ceramics were examined over a temperature range spanning from −100 to 400 °C and at frequencies from 1 kHz to 1 MHz (Figure S7, Supporting Information). The BT‐SMT‐*x*NBT ceramics have unique frequency dispersion phenomena and single dielectric peaks (*T*_m_), indicative of enhanced relaxor behavior. It can also be observed that the *T*_m_ gradually shifts toward higher temperatures, which may be related to the distortion of the octahedral [TiO_6_] in the Raman spectra. When the NBT content increases to 0.2, the *T*_m_ approaches room temperature (**Figure** **3**a), which facilitates the development of polymorphic heterogeneous structures, thereby improving polarization. Concurrently, the dielectric constant progressively increases with the introduction of NBT content, resulting in a larger polarization response under the same electric field, a desirable trait for achieving high polarization. Nevertheless, an excessively high dielectric constant can induce a pronounced electrostriction effect, potentially compromising the breakdown strength.^[^ ^23^ ^]^ In comparison to other samples, the BT‐SMT‐0.2NBT ceramics possess a moderate dielectric constant, demonstrating a high breakdown strength and large polarization. Furthermore, the dielectric constant of BT‐SMT‐0.2NBT_RRP_ ceramics displays minimal variation between 20 and 150 °C (Figure S7f, Supporting Information). This smaller rate of change in the dielectric constant suggests that the polarization properties of the material remain more stable at high temperatures, further contributing to its high‐temperature energy storage capabilities.^[^ ^57^ ^]^ To quantify the degree of relaxation, a modified Curie‐Weiss law formula was employed for fitting,^[^ ^58^ ^]^ yielding *γ* values consistently above 1.5 (Figure 3a), a clear indication of relaxor behavior. The remarkable temperature stability of the BT‐SMT‐0.2NBT_RRP_ ceramic can be attributed to its internal structure, which is insensitive to temperature fluctuations. The quantity and relative strength of the XRD diffraction peaks remain constant in the tested temperature range (≈25–205 °C) (Figure S8, Supporting Information). The (111) and (200) diffraction peaks shift to lower angles with increasing temperatures (Figure 3b), which may be attributed to the thermal expansion of the crystal lattice. The absence of diffraction peak splitting throughout the temperature range, underscoring the stable phase structure that is instrumental in facilitating a broad operating temperature range for the ceramics.

### 2.4 Elucidation of Breakdown Behavior Optimization

A dense microstructure and fine grain size are prerequisites for obtaining high *BDS*. The BT‐SMT‐*x*NBT ceramics are noted for a dense microstructure without any impurities (Figure S9a–e, Supporting Information), as corroborated by the XRD data. The average grain size initially decreases and then increases with the introduction of NBT (Figure S9f, Supporting Information). When NBT is introduced in greater quantities, the volatilization of Na^+^ and Bi^3+^ during sintering intensifies, resulting in the generation of oxygen vacancies that promote the growth of ceramic grains.^[^ ^45^, ^59^ ^]^ To elucidate the mechanism by which microstructural refinement enhances breakdown strength, SEM images of BT‐SMT‐0.2NBT and BT‐SMT‐0.2NBT_RRP_ (Figure S10, Supporting Information) ceramics were used as representative models. Finite element calculations were performed employing MATLAB and COMSOL software to simulate the dynamics of electric field and potential distribution when subjected to a field of 700 kV·cm^−1^ (Figure 3c‐f). In the case of BT‐SMT‐0.2NBT ceramics, the propagation of electric trees is accompanied by the continuous distortion and extension of the equipotential line toward the upper boundary. This process culminates in the electrical tree penetrating the entire area, ultimately resulting in breakdown (Figure 3c,e). In sharp contrast, under an identical electric field, the potential drop rate of BT‐SMT‐0.2NBT_RRP_ ceramics is dramatic, and the distortion of equipotential lines becomes progressively ambiguous over time. As a result, the propagation of electrical tree is curtailed, covering only 3/4 of the distance (Figure 3d,f). This is attributed to the higher dielectric constant of the grains compared to grain boundaries, which allows the latter to withstand higher electric fields, thereby impeding the propagation of electric trees.^[^ ^33^, ^60^ ^]^ The RRP reduces grain size and concurrently increases the density of grain boundaries, which can effectively obstruct the propagation of electrical trees and hence shorten the electrical tree propagation path and boost breakdown strength. The outcomes of the finite element simulations closely align with the empirical test results, confirming that the reduction in grain size is beneficial to improving the breakdown strength and consequently enhancing the energy storage performance of lead‐free ceramics.

### 2.5 Analysis of Composition‐Driven Relaxation Characteristics

**Figure 4:**
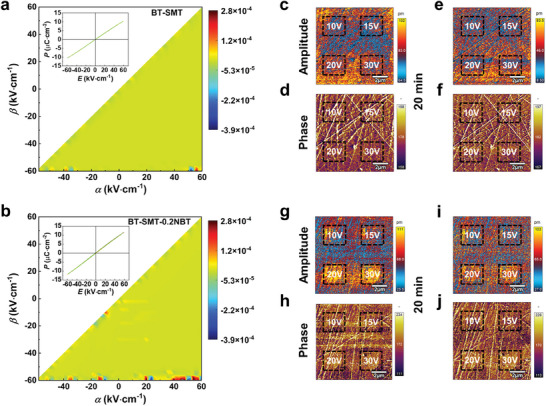
a, b) Evolution of the FORC distribution of BT‐SMT and BT‐SMT‐0.2NBT ceramics, and the inset shows the FORC loops of BT‐SMT and BT‐SMT‐0.2NBT ceramics. PFM amplitude and phase images with different voltages and relaxation durations: c–f) BT‐SMT and g–j) BT‐SMT‐0.2NBT.

The first‐order reversal curve (FORC) distribution was employed to analyze the polarization behavior of the BT‐SMT‐*x*NBT ceramics. In this study, the FORC distribution of the ceramics was derived from a series of FORC loop measurements. The evolution of these diagrams (**Figure** **4**a) reveals that the BT‐SMT ceramic sample maintains a uniform distribution in the entire range of electric fields, indicative of enhanced relaxor behaviors. This is ascribed to the diminished FE nonlinearity and dispersed polarization contribution.^[^ ^61^ ^]^ In the centroid region, the BT‐SMT‐0.2NBT ceramics have heightened FORC intensity compared to the BT‐SMT ceramics, signifying a more robust polarization intensity (Figure 4b). This phenomenon is related to domain switching and domain wall movement at low electric fields, which in turn contributes to the increase in dielectric constant and polarization of the material. Notably, the high‐intensity zones of the BT‐SMT‐0.2NBT ceramics contract toward the origin point. This result demonstrates that the BT‐SMT‐0.2NBT ceramics possess dielectric relaxation behavior, characterized by reduced coercive fields and attenuated nonlinearity.^[^ ^23^ ^]^ The energy storage performance is intricately connected to the dynamic response of domain structures to external electric fields. To address this, the dynamic domain responses of the BT‐SMT, BT‐SMT‐0.2NBT, and BT‐SMT‐0.4NBT were probed using Piezo‐response Force Microscopy (PFM) (Figure 4c–j and Figure S11, Supporting Information). When the same voltage is applied, the PFM signal intensifies with increasing NBT content, reflecting augmented polarization that aligns with the bipolar *P*‐*E* loop measurements. The PFM signal is hard to pick up in BT‐SMT ceramic when the loading voltage is low (Figure 4c,d). This indicates that BT‐SMT ceramics have minimal polarization dipoles, and the polarization mechanism is primarily governed by ion displacement polarization, which is consistent with the findings of FORC.^[^ ^62^, ^63^ ^]^ Polarization behavior in these ceramics can only be induced at higher electric fields, resulting in reduced saturation polarization intensity at the macro level, thus hindering the achievement of high energy storage density. Moreover, upon the removal of applied voltage for 20 min, majority of the domains in BT‐SMT‐0.2NBT (Figure 4i,j) were capable switching back to their original state. However, the BT‐SMT‐0.4NBT ceramic still retained the obvious electrical signals (Figure S11, Supporting Information), suggesting that excessive NBT content significantly increases the domain size of BT‐SMT‐0.4NBT ceramics, leading to significant polarization hysteresis, which is not conducive to achieving high energy storage efficiency. In light of these findings, optimization of doping levels is paramount in maintaining reasonable domain size, thereby contributing to the attainment of low hysteresis and high polarization characteristics, which are essential for the development of high‐performance lead‐free dielectric ceramics.

### 2.6 Local Polymorphic Heterogeneous Structures and Atomic Polar Displacement

**Figure 5:**
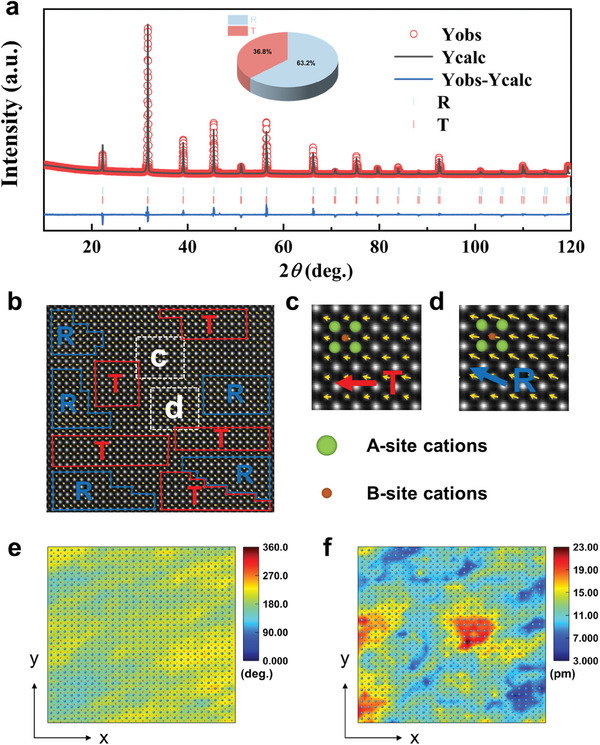
a) Rietveld refinement analysis of the BT‐SMT‐0.2NBT ceramic. The inset illustrates the proportional distribution of the phases. b) Atomic‐resolution HAADF‐STEM polarization vector image along [100] direction. c,d) Magnification of the marked areas. e) Polarization angle mapping, and f) Polarization magnitude mapping. The light green and orange spheres represent A‐site and B‐site cations, respectively. The yellow arrows show the B‐site cation displacement vectors in each unit cell.

To unveil the intrinsic origin of the exceptional comprehensive performance of BT‐SMT‐0.2NBT ceramics, an in‐depth analysis of the phase structure was conducted using Rietveld refinement (**Figure** **5**a). The *R*_wp_ and *X* ^2^ evaluation parameters demonstrate strong reliability, as both are notably below 10% (Table S2, Supporting Information). The analysis confirms the concurrent presence of R3m (63.2%) and P4mm (36.8%) phases in the BT‐SMT‐0.2NBT ceramic. The dominance of the strongly polar R phase facilitates an increase in electric field‐induced polarization within the system. To delve into the atomic scale microstructures, high‐angle annular dark field (HADDF) was used further to explore the local structure of the BT‐SMT‐0.2NBT ceramic, and proprietary software was used to calculate the polarization displacement vector. The lattice fringes (Figure S12, Supporting Information), reveal that the BT‐SMT‐0.2NBT ceramic exhibit a good crystalline quality with a pure perovskite structure, being consistent with the XRD results. The A‐site (Ba/Sr/Bi/Na) cations are more prominently visible in the HAADF image due to their higher average atomic number over the B‐site (Ti/Mg) cations. A custom MATLAB program was engaged to precisely fit the atomic column occupancy using a 2D Gaussian function. The magnitude and direction of the polarization vector were determined based on the offset of the center of the B‐site column relative to the centers of its four neighboring A‐site columns, as indicated by yellow arrows (Figure 5b). The results reveal that the BT‐SMT‐0.2NBT ceramic experiences a wide range of local random fluctuations in the [100]_c_ direction, with the polarization vectors oriented close to the [001]_c_ direction indicative of the T‐phase (Figure 5c), while those aligned along the [011]_c_ direction correspond to the R‐phase (Figure 5d).^[^ ^12^, ^24^, ^64^ ^]^ The presence of local T‐phase and R‐phase is further corroborated by the polarization vector in the [100]c direction, as illustrated in Figure 5b, which is consistent with the refined XRD results. On the one hand, the domain‐switching barriers and polarization anisotropy in the coexistence of R and T phases structure are further diminished, leading to a smoother domain‐switching pathway, and rendering the polarization direction more susceptible to change under an external electric field. It is noted that this field‐induced phase transition is reversible, thus facilitating the achievement of a higher *P*_max_ and a lower *P*_r_.^[^ ^22^, ^34^, ^65^ ^]^ On the other hand, the formation of the polymorphic heterostructure can enhance the stability of local structures during temperature variations, thereby contributing to the improvement of energy storage stability in a broad temperature range.^[^ ^11^ ^]^ Furthermore, there are apparently alternate distributions of local domain structure, where heavily ratio of R phase nanodomain is observed to be embedded in the T phase nanodomain, as further evidenced by the polarization angle and magnitude of BT‐SMT‐0.2NBT ceramics along the [100] orientation (Figure 5e,f), being consistent with the result of refinement structure (see Figure 5a).

## 3 Conclusion

In summary, a giant *W*_rec_ of 10.1 J·cm^−3^ and an ultra‐high *η* of 95.0% were concurrently achieved in BT‐SMT‐0.2NBT_RRP_ ceramics via a multi‐scale synergistic strategy. This strategy capitalizes on the local polymorphic heterogeneous structures and repeated rolling process, which contributes to reduced hysteresis and increased polarization strengths, respectively. More encouragingly, the ceramics by rational design are endowed with remarkable high‐temperature energy storage capabilities (*W*_rec_ = 7.2 J·cm^−3^, *η* = 92.2%, 150 °C) and temperature‐insensitivity (*W*_rec_ ≈ 5.8 ± 0.2 J·cm^−3^, *η* ≈ 95.4 ± 4.0%, ≈20–150 °C). These attributes underscore that this research provides a paradigm for the development of lead‐free dielectric ceramics with outstanding energy storage performance across a broad‐high temperature, catering to the pressing requirements of next‐generation environmentally friendly dielectric capacitors.

## Conflict of Interest

The authors declare no conflict of interest.

## Supporting information



Supporting Information

## Data Availability

The data that support the findings of this study are available from the corresponding author upon reasonable request.
