# Supplementary material for: A Broad‐High Temperature Ceramic Capacitor with Local Polymorphic Heterogeneous Structures
Source: Adv Sci (Weinh). 2024 Oct 30;12(1):2409814. doi: 10.1002/advs.202409814 (PMC11714191; doi:10.1002/advs.202409814)
Supplement: Supplementary file 1 — Supporting Information [file ADVS-12-2409814-s001.docx]

Supporting Information

**A Broad-High Temperature Ceramic Capacitor with Local Polymorphic Heterogeneous Structures**

*Binglong Zheng, Ying Lin,* Haibo Yang,* Hongmei Jing,* Hu Nan, Yifei Wang, Fang-Zhou Yao, Minquan Wang, Qibin Yuan**

Dr. B. Zheng, Prof. Y. Lin, Prof. H. Yang, Dr. M. Wang

Shaanxi Key Laboratory of Green Preparation and Functionalization for Inorganic Materials

School of Materials Science and Engineering

Shaanxi University of Science and Technology

Xi’an 710021, China

E-mail: linying@sust.edu.cn

E-mail: yanghaibo@sust.edu.cn

Dr. H. Jing

School of Physics and Information Technology

Shaanxi Normal University

Xi’an 710119, China

E-mail: jhmei.dengdai@snnu.edu.cn

Dr. H. Nan

School of Microelectronics

Faculty of Electronic and Information Engineering

Xi’an Jiaotong University

Xi’an 710049, China

Prof. Y. Wang

State Key Laboratory for Mechanical Behavior of Material

School of Materials Science and Engineering

Xi’an Jiaotong University

Xi’an, 710049, China

Prof. F-Z. Yao,

Research Center for Advanced Functional Ceramics

Wuzhen Laboratory

Jiaxing 314500, China

Prof. Q. Yuan

School of Electronic Information & Artificial Intelligence

Shaanxi University of Science and Technology

Xi’an 710021, China

E-mail: yuanqibin-sust@163.com

**Experimental Section**

**Sample preparation:** The lead-free ceramics of (1-*x*)[0.92BaTiO_3_-0.08Sr(Mg_1/2_Ti_3/4_)]-*x*(Na_0.5_Bi_0.5_)TiO_3_ (abbreviated as BT-SMT-*x*NBT, where *x*=0.1, 0.2, 0.3, 0.4) were prepared by the solid-state reaction method. The dried raw powders of BaCO_3_ (99%), TiO_2_ (98%), SrCO_3_ (99%), MgO (99.9%), Na_2_CO_3_ (99.8%) and Bi_2_O_3_ (99%) (Sinopharm Chemical Reagent Co., Ltd) were used as the starting materials and were stoichiometrically weighed and then mixed in ethanol for 24 h, except that Bi_2_O_3_ was over-added by 2%. The powders underwent calcination at 850 ℃ for 4 hours followed by 24 hours of ball milling. Subsequently, the dried powders from the second milling were cold isostatically pressed at 200 MPa for 5 minutes to produce green bodies measuring 12 mm in diameter. The samples were obtained by sintering green bodies at 1140 ℃~1230 ℃ for 3~4 h. In addition, The ceramic tapes based on BT-SMT-0.2NBT were fabricated using a repeated rolling processing (RRP) method, starting from pre-sintered powders. The calcined BT-SMT-0.2NBT powders were mixed thoroughly with 25 wt.% polyvinyl alcohol (PVA), followed by repeated rolling. Subsequent removal of PVA at 600 ℃ enabled the production of BT-SMT-0.2NBT_RRP_ ceramics through sintering at 1085 ℃~1100 ℃ for 2 hours.

**Structural characterization:** The X-ray diffraction (XRD, D8 Advanced, Bruker, Germany) with Cu Kα was used to measure the phase structure. The Raman spectras were obtained by Raman scattering spectrometer (Horiba Jobin-Yvon HR800, HORIBA Jobin Yvon, France). The microstructure of the samples was characterized using scanning electron microscopy (SEM, S-4800, Hitachi, Japan), and the grain size was determined with Nano Measurer. Atomic-scale images were obtained utilizing an atomic-resolution high-angle annular dark-field scanning transmission electron microscope(HAADF-STEM, Titan Themis G2 microscope, FEI, USA), followed by processing through 2D Gaussian fitting in MATLAB scripts to analyze the polarization vector, magnitude, and angle maps.

**Measurement of electrical properties:** The dielectric properties of the silver electrode specimens were assessed utilizing an LCR analyzer (E4980A, Agilent, USA) over a temperature range from -100 °C to 400 °C at a heating rate of 3 °C/min, and across frequencies ranging from 1 kHz to 1 MHz. The ceramic was reduced to a thickness of 30~50 mm, followed by the fabrication of gold electrodes with a 1.5 mm diameter using ion sputtering. The *P*-*E* loop measured by a ferroelectric analyzer (Premier II, Radiant, USA) was used to evaluate the energy storage performance. The charge/discharge performance of samples with a thickness of 60 µm was performed using a commercial charge-discharge device (CFD-001, Gogo Instruments Technology, China). The dynamic response of the domain to the external electric fields was investigated by piezoelectric force microscopy (PFM, MFP-3D, Asylum Research, USA). The samples were well polished using diamond suspension before PFM measurements.

**Results and Discussion**


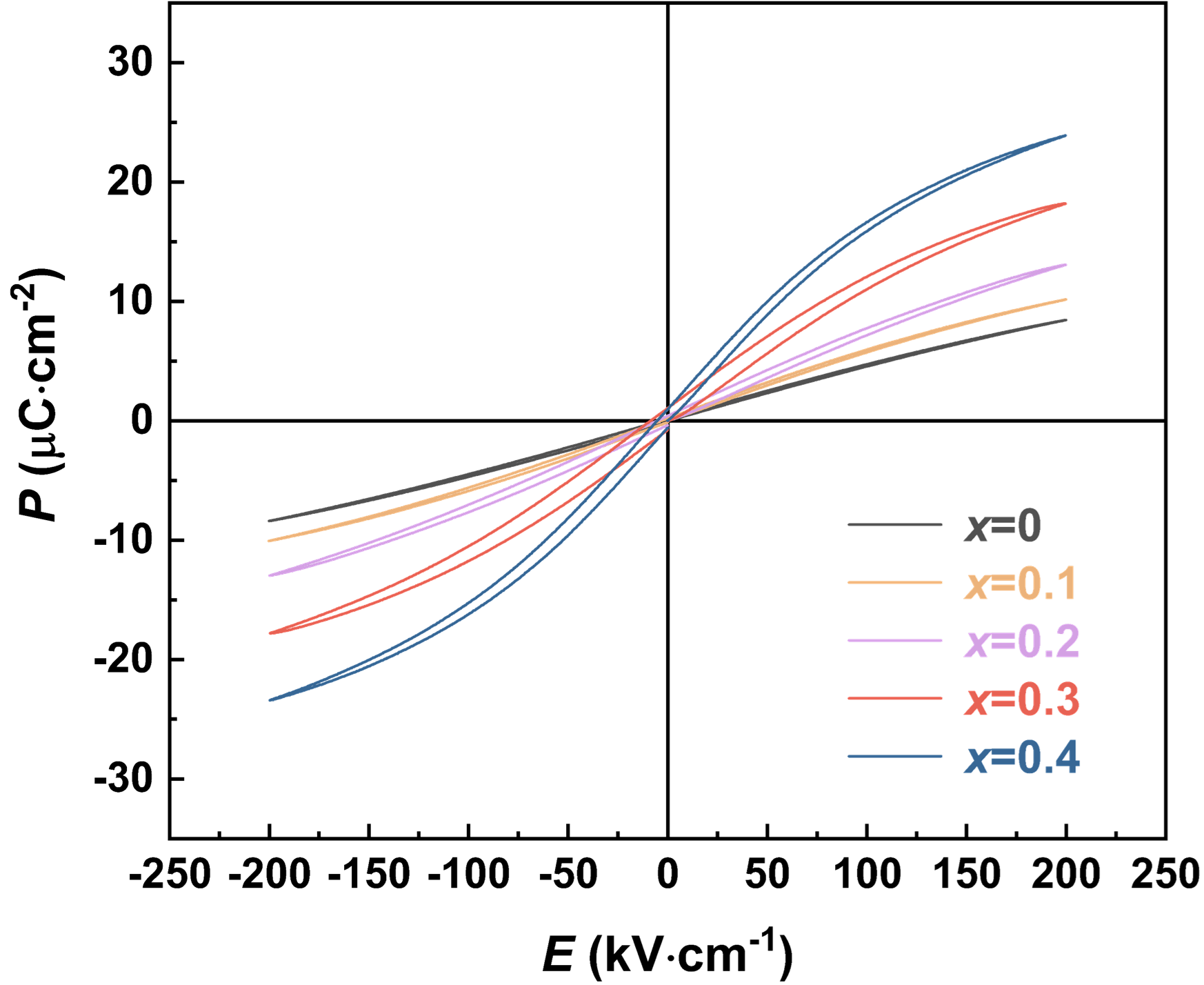


**Figure S1.** Bipolar *P*-*E* loops at 200 kV·cm^-1^ of the BT-SMT-*x*NBT ceramics (*x*=0, 0.1, 0.2, 0.3, 0.4).





**Figure S2.** Weibull distribution of theoretical *BDS* for BT-SMT-0.2NBT_RRP_ ceramic.


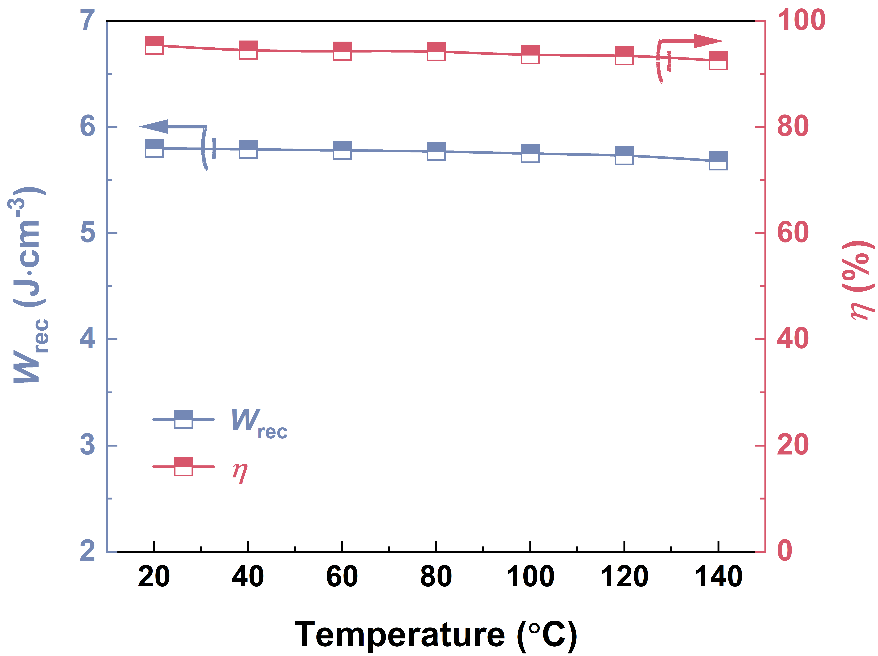


**Figure S3.** *W*_rec_ and *η* values of the BT-SMT-0.2NBT_RRP_ ceramic under 400 kV·cm^-1^ at various temperatures.


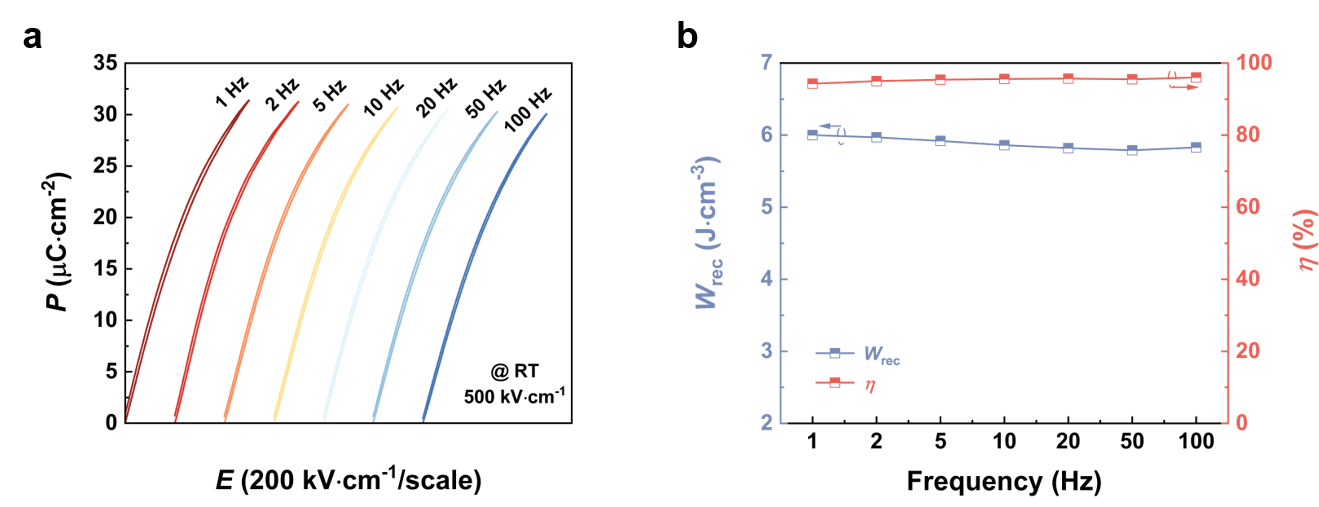


Figure S4. Frequency-dependent *P*-*E* loops of BT-SMT-0.2NBT_RRP_ ceramic at room temperature under 500 kV cm^-1^. (d) *W*_rec_ and *η* as a function of frequency under 500 kV cm^-1^.


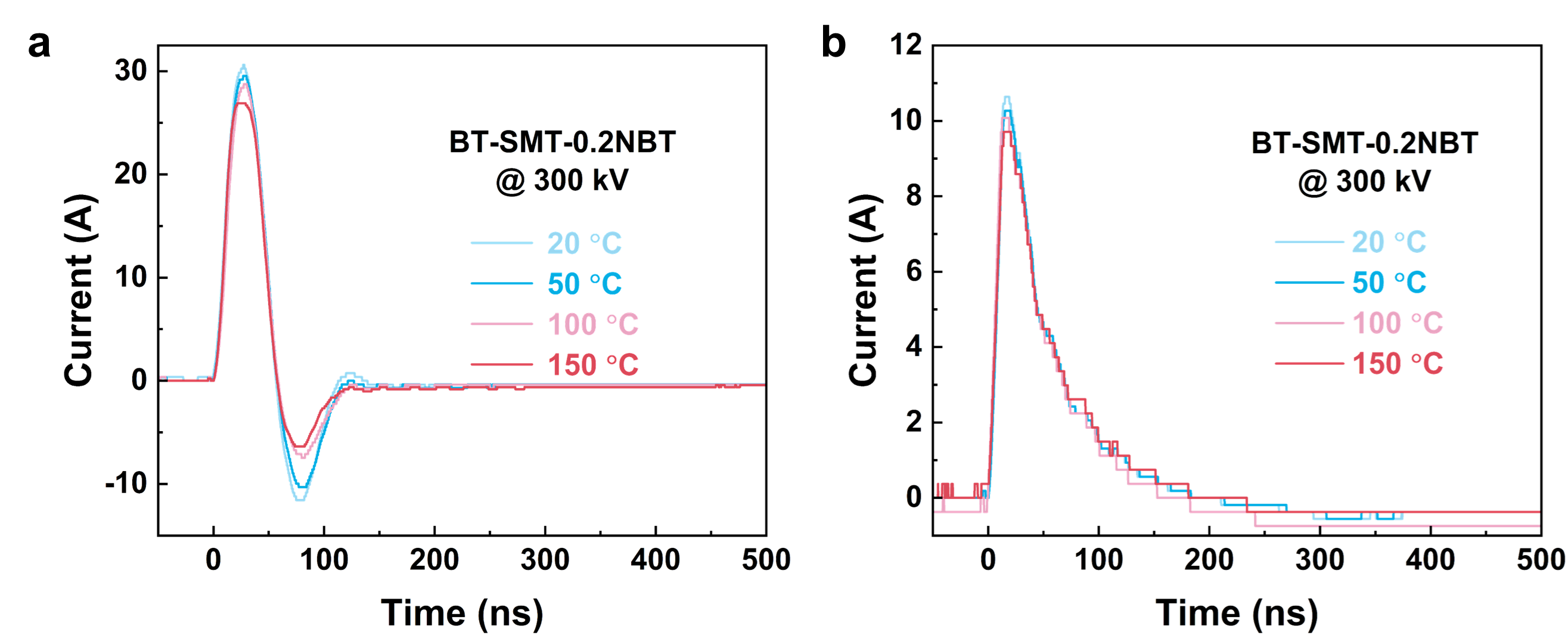


**Figure S5.** a) Under-damped discharge waveforms and b) over-damped discharge waveforms for BT-SMT-0.2NBT_RRP_ ceramic at different temperatures.


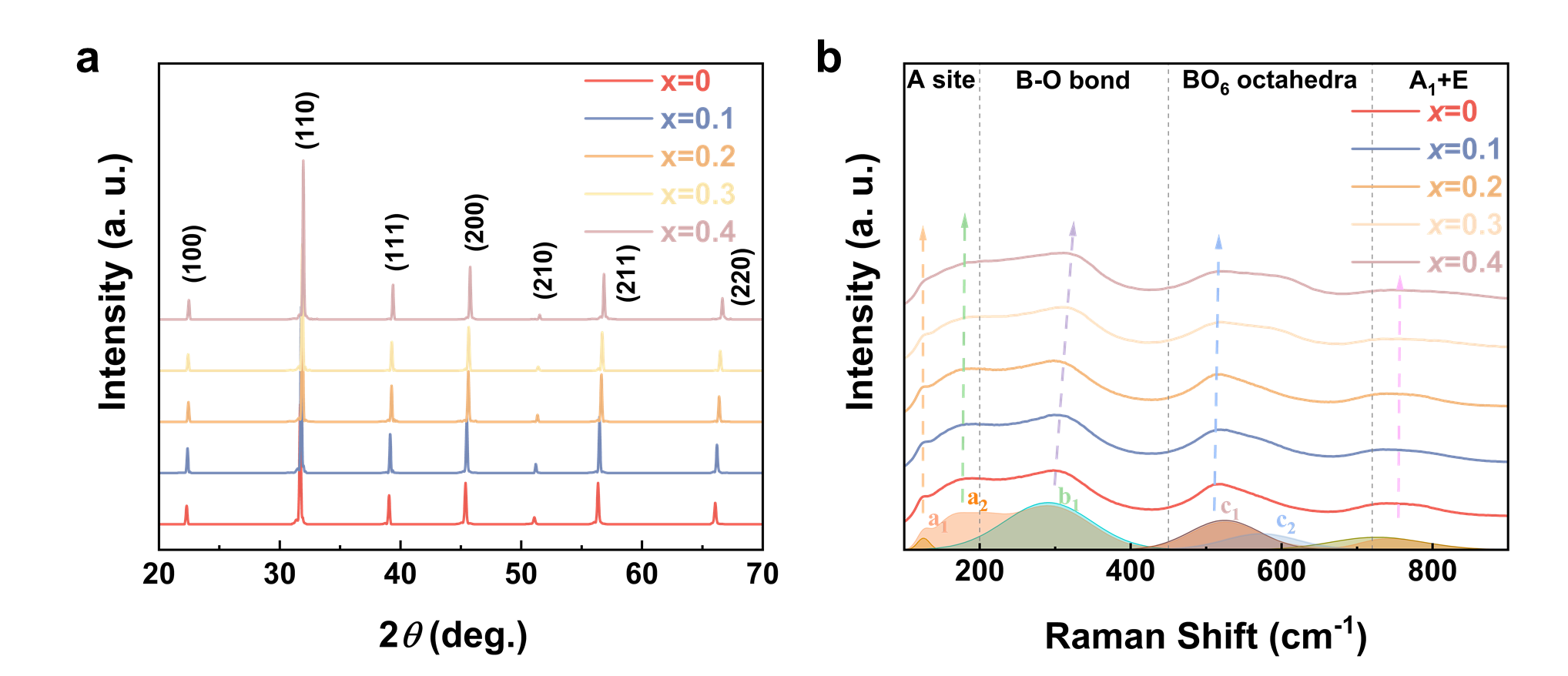


**Figure S6.** a) XRD patterns of BT-SMT-*x*NBT ceramics. b) Raman spectra of BT-SMT-*x*NBT ceramics.


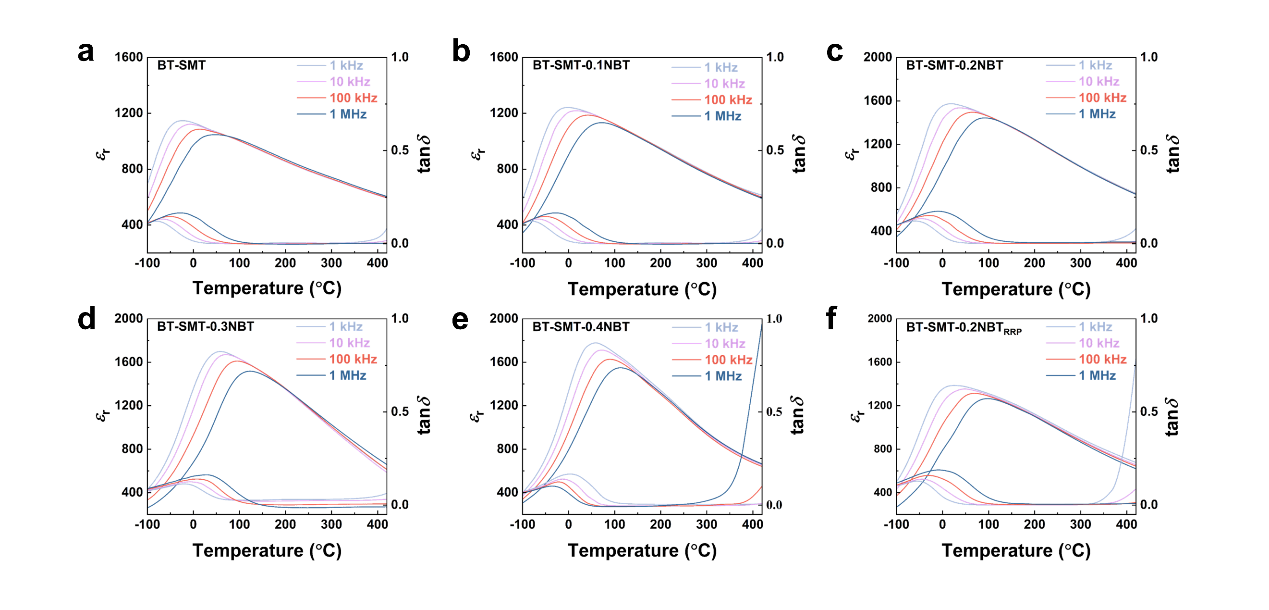


**Figure S7.** Temperature dependence of the dielectric permittivity (*ε*_r_) and dielectric loss (tan*δ*) at 1 kHz~1 MHz for the BT-SMT-*x*NBT ceramics from -100 ℃ to 400 ℃: a) *x*=0; b) *x*=0.1; c) *x*=0.2; d) *x*=0.3; e) *x*=0.4.

The dielectric permittivity at 1 kHz can be analyzed with the Curie-Weiss law as follows:

$\frac{1}{\varepsilon_{\text{r}}}-\frac{1}{\varepsilon_{\text{m}}}=\frac{\left(T-T_{m}\right)^{\gamma}}{C}$ (1)

where *γ* designs the diffuseness degree and C represents the Curie constant.


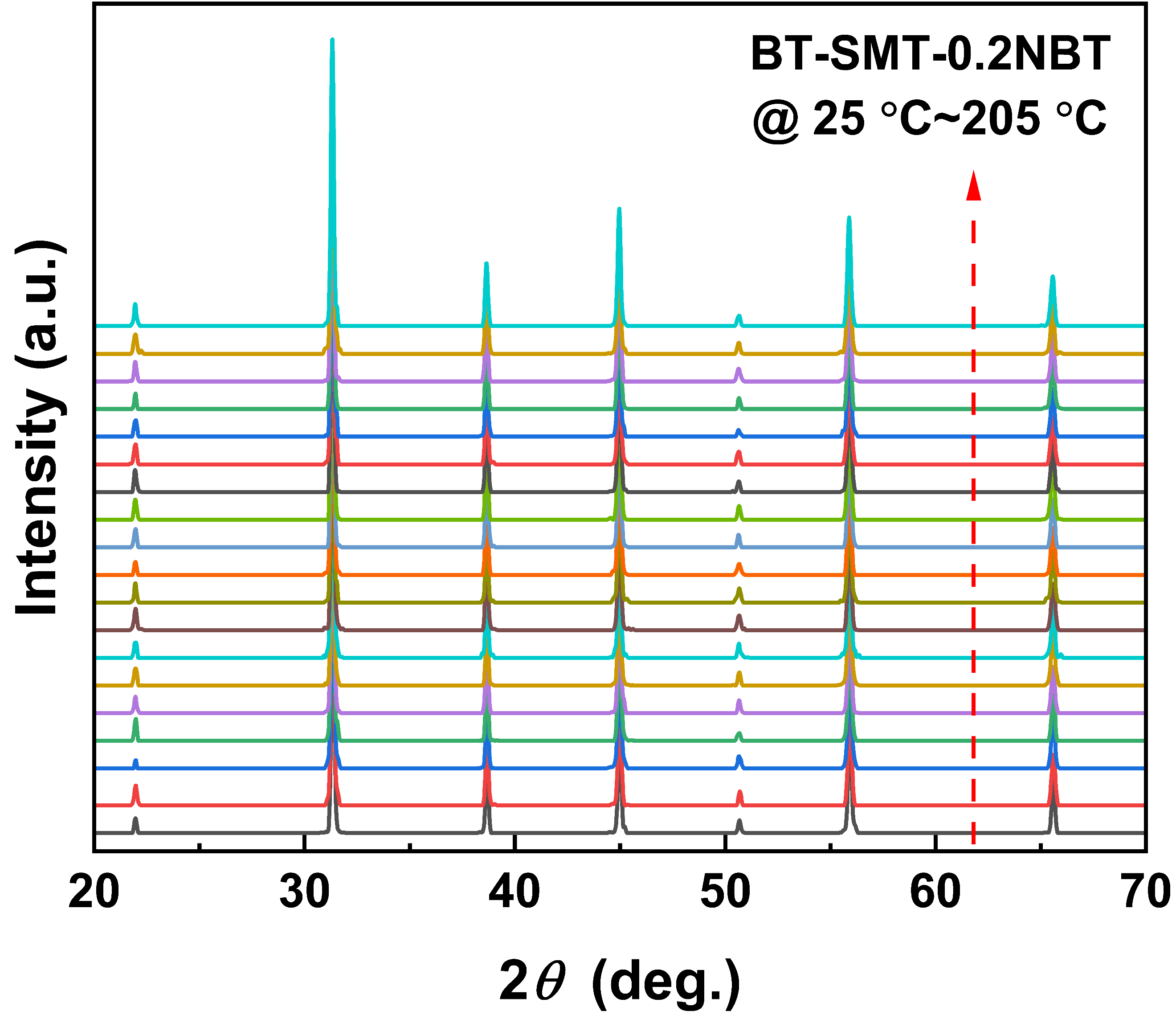


**Figure S8.** Temperature-dependent XRD patterns of the BT-SMT-0.2NBT ceramic from 25 ℃ to 205 ℃.


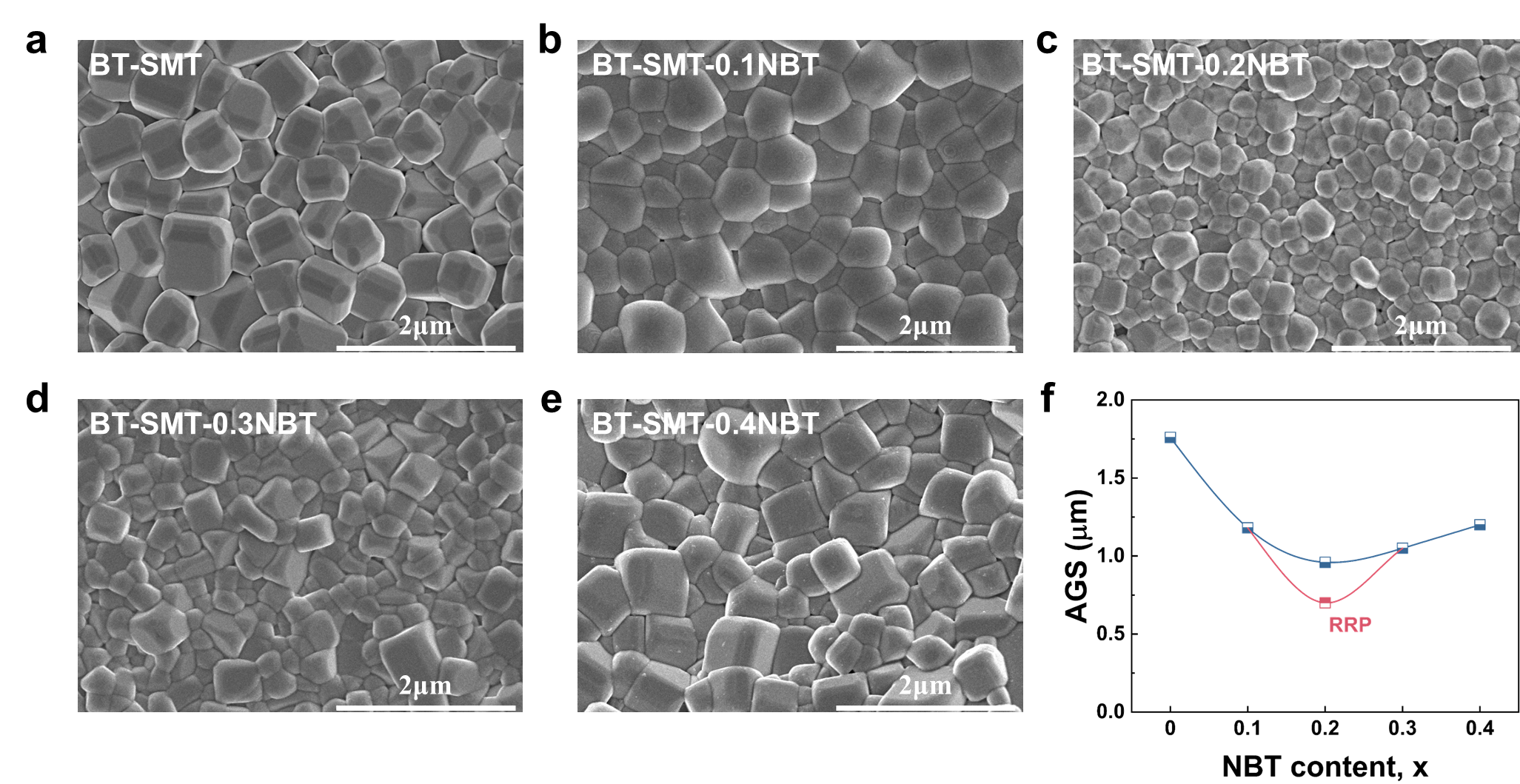


**Figure S9.** SEM images of a~e) *x*=0, 0.1, 0.2, 0.3, 0.4. f) Variation of average grain size of BT-SMT-*x*NBT ceramics.


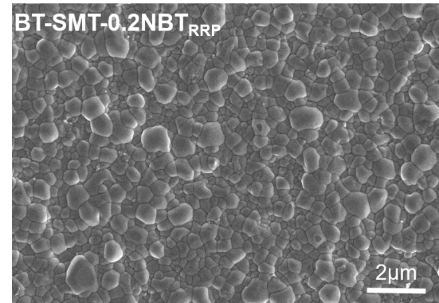


**Figure S10.** SEM images of BT-SMT-0.2NBT_RRP_ ceramic.

The simulated breakdown model was established by formula through the finite element method^[1]^:

**** (2)

in which *p* represents the probability of electrical tree development, while (*x'*, *y'*) and (*x*, *y*) denote the coordinates of non-breakdown and breakdown positions. The threshold of electric potential is denoted by $\varphi$_0_, and the fractal dimension (*n*) is set to 3 for this simulation. Among the terms in the right polynomial, the first one signifies the direction of electrical dendrite growth, the second one mirrors the growth difficulty of electrical trees, while the final term is linked to the material's dielectric properties, with *α*, *β*, and *γ* serving as weighting coefficients.

The FORC distribution is derived from a series of FORC loops and the Preisach density *ρ*(*α*, *β*) is calculated by the following equation^[2, 3]^:

$\rho\left(\alpha,\beta\right)=\frac{1}{2}\frac{\partial P^{2}\left(\alpha,\beta\right)}{\partial\alpha\partial\beta}$ (3)

where *ρ*(*α*, *β*) is the polarization of the FORC loop, *α* is the reversal electric field, and *β* is the actual electric field. The Preisach density represents the density distribution of the ideal ‘hysteron’ and provides information on the local switching behavior. In this work, we set *E*_max_=60 kV∙cm^-1^, ∆*α*=∆*β*=4 kV∙cm^-1^ and FORC loops were measured.


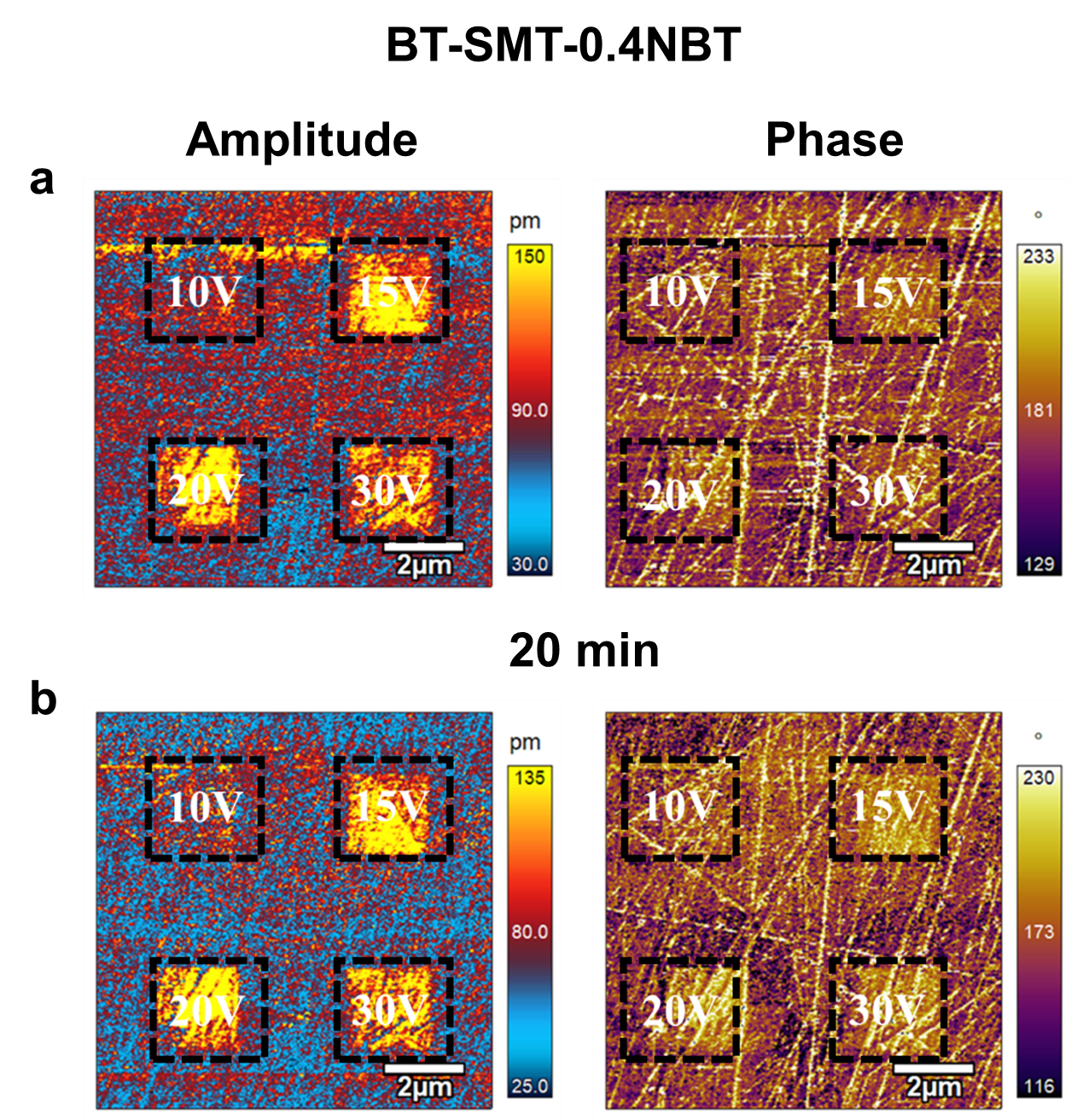


**Figure S11.** a, b) Out-of-plane PFM phase images along with amplitude after polarization with different voltages and relaxation durations of BT-SMT-0.4NBT ceramic.


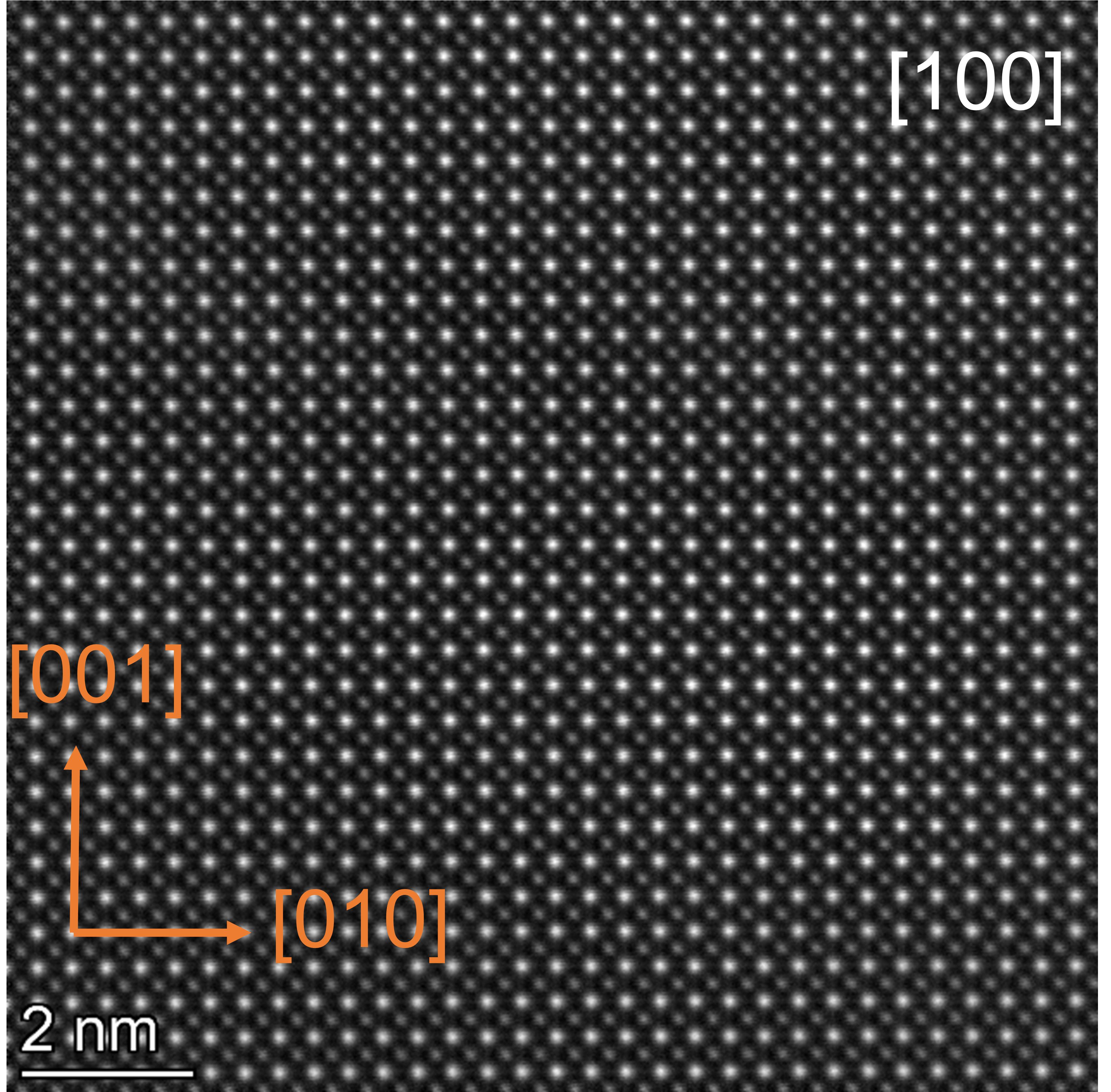


**Figure S12.** Lattice fringes along [100]_c_ of BT-SMT-0.2NBT ceramic.

**Table S1.** Comparison of the *W*_rec_, *η* and *BDS* between this work and previous reports.

| Compositions | *W*_rec_ (J·cm^-3^) | | *η* (%) | | *BDS* (kV·cm^-1^) | | | Ref |  |
| --- | --- | --- | --- | --- | --- | --- | --- | --- | --- |
|  | RT | ~150 ℃ | RT | ~150 ℃ | | RT | ~150 ℃ |  |  |
| BT-NBT-NN | 10.59 | 4.97 | 87 | 92 | | 563 | 350 | [4] |  |
| BT-BMT | 9.03 | 6.46 | 95 | 93 | | 720 | 600 | [5] |  |
| BBT | 10.10 | 3.35 (120 ℃) | 90 | 91 (120 ℃) | | 700 | 300 | [6] |  |
| BT-NBT-CZ | 9.04 | 3.27 (200 ℃) | 87 | 92 (200 ℃) | | 540 | 300 | [7] |  |
| BSBT-CT (VPP) | 4.00 | 3.01 | 80 | 72 | | 480 | 400 | [8] |  |
| BST-BMZ (VPP) | 10.30 | 6.00  (120 ℃) | 88 | 95  (120 ℃) | | 720 | 450 | [9] |  |
| BNTFN | 13.8 |  | 82 |  | |  |  | [10] |  |
| BNT-SBT-LMT | 7.30 | 3.76 (200 ℃) | 93 | 89 (200 ℃) | | 390 | 260 | [11] |  |
| BNT-SNA | 6.64 | 3.28 | 96 | 92 | | 520 | 300 | [12] |  |
| BNST-BMZ | 8.46 | 4.74 (200 ℃) | 86 | 79 (200 ℃) | | 522 | 300 | [13] |  |
| KNN-H | 10.06 | 3.18 (140 ℃) | 91 | 80 (140 ℃) | | 740 | 400 | [14] |  |
| KNN-BNZ | 8.09 |  | 88 |  | | 870 |  | [15] |  |
| NN-BNT-CT | 7.10 | 3.50 (160 ℃) | 90 | 80 (160 ℃) | | 646 | 450 | [16] |  |
| NN-BLT | 8.73 | 3.60 (200 ℃) | 80 | 90 (200 ℃) | | 485 | 380 | [17] |  |
| BKT-BT-NN | 7.57 | 4.06 (200 ℃) | 81 | 80 (200 ℃) | | 460 | 300 | [18] |  |
| BT-SMT-NBT | 10.12 | 7.18 | 95 | 92 | | 820 | 640 | This work | |

**Table S2.** Refined structural parameters by using the Rietveld method for the BT-SMT-0.2NBT.

| Space group | Lattice parameters | Cell volume  (Å^3^) | *R*_wp_  (%) | *R*_p_  (%) | χ^2^ |
| --- | --- | --- | --- | --- | --- |
| R3m (63.21%) | a=b=c=3.99076 Å, *α*=*β*=*γ*=89.98099° | 63.557 | 9.33 | 5.65 | 9.14 |
| P4mm (36.79%) | a=b=3.99439 Å, c=3.99076 Å, *α*=*β*=*γ*=90° | 63.790 |  |  |  |

**References**

1. Q. B. Yuan, F.-Z. Yao, S.-D. Cheng, L. X. Wang, Y. F. Wang, S.-B. Mi, Q. Wang, X. H. Wang, H. Wang, *Adv. Funct. Mater.* **2020**, *30*, 200191.
2. H. Pan, J. Ma, J. Ma, Q. H. Zhang, X. Z. Liu, B. Guan, L. Gu, X. Zhang, Y.-J. Zhang, L. L. Li, Y. Shen, Y.-H. Lin, C.-W. Nan, *Nat. Commun.* **2018**, *9*, 1813.
3. M. Q. Wang, Y. Lin, M. Chen, M. Zhang, Q. B. Yuan, H. B. Yang, *J. Mater. Chem. C* **2023**, *11*, 6407.
4. L. Chen, N. Wang, Z. F. Zhang, H. F. Yu, J. Wu, S. Q. Deng, H. Liu, H. Qi, J. Chen, *Adv. Mater.* **2022**, *34*, 2205787.
5. C. B. Long, W. J. Zhou, H. M. Song, K. Zheng, W. Ren, H. J. Wu, X. D. Ding, L. J. Liu, *Acta Mater.* **2023**, *256*, 119135.
6. Z. Sun, J. Zhang, H. J. Luo, Y. H. Yao, N. Wang, L. Chen, T. Y. Li, C. Z. Hu, H. Qi, S. Q. Deng, L. C. Gallington, Y. P. Zhang, J. C. Neuefeind, H. Liu, J. Chen, *J. Am. Chem. Soc.* **2023**, *145*, 6194.
7. L. Chen, F. Li, B. T. Gao, C. Zhou, J. Wu, S. Q. Deng, H. Liu, H. Qi, J. Chen, *Chem. Eng. J.* **2023**, *452*, 139222.
8. Y. Li, M.-Y. Tang, Z.-G. Zhang, Q. Li, J.-L. Li, Z. Xu, G. Liu, F. Li, *Rare Metals* **2023**, *42*, 1261.
9. W. Wang, L. Y. Zhang, C. Li, D. O. Alikin, V. Y. Shur, X. Y. Wei, F. Gao, H. L. Du, L. Jin, *Chem. Eng. J.* **2022**, *446*, 137389.
10. L. Chen, H. F. Yu, J. Wu, S. Q. Deng, H. Liu, L. F. Zhu, H. Qi, J. Chen, *Nano-Micro Lett.* **2023**, *15*, 65.
11. R. R. Kang, Z. P. Wang, M. Wu, S. D. Cheng, S. B. Mi, Y. H. Hu, L. X. Zhang, D. Wang, X. J. Lou, *Nano Energy* **2023**, *112*, 108477.
12. F. Yan, G. L. Ge, J. Qian, J. F. Lin, C. K. Chen, Z. F. Liu, J. W. Zhai, *Small* **2022**, *19*, 2206125.
13. X. P. Zhu, Y. F. Gao, P. Shi, R. R. Kang, F. Kang, W. J. Qiao, J. Y. Zhao, Z. Wang, Y. Yuan, X. J. Lou, *Nano Energy* **2022**, *98*, 107276.
14. L. Chen, S. Q. Deng, H. Liu, J. Wu, H. Qi, J. Chen, *Nat. Commun.* **2022**, *13*, 3089.
15. M. Zhang, H. B. Yang, Y. Lin, Q. B. Yuan, H. L. Du, *Energy Storage Mater.* **2022**, *45*, 861.
16. S. Y. Wu, B. Fu, J. J. Zhang, H. W. Du, Q. Zong, J. Y. Wang, Z. B. Pan, W. F. Bai, P. Zheng, *Small* **2023**, *19*, 2303915.
17. A. W. Xie, R. R. Zuo, Z. L. Qiao, Z. Q. Fu, T. F. Hu, L. F. Fei, *Adv. Energy Mater.* **2021**, *11*, 2101378.
18. L. Chen, F. X. Long, H. Qi, H. Liu, S. Q. Deng, J. Chen, *Adv. Funct. Mater.* **2021**, *32*, 2110478.
